# Natural compounds in the management of polycystic ovary syndrome: a comprehensive review of hormonal regulation and therapeutic potential

**DOI:** 10.3389/fnut.2025.1520695

**Published:** 2025-02-11

**Authors:** Jingyi Yuan, Zhenmin Li, Yongjiang Yu, Xiuge Wang, Yunyun Zhao

**Affiliations:** ^1^College of Chinese Medicine, Changchun University of Chinese Medicine, Changchun, Jilin, China; ^2^Department of Endocrine and Metabolic Disease, The Affiliated Hospital to Changchun University of Chinese Medicine, Changchun, Jilin, China

**Keywords:** polycystic ovary syndrome, natural compounds, insulin resistance, hormone regulation, therapeutic strategies

## Abstract

Polycystic ovary syndrome (PCOS) is a multifaceted endocrine disorder characterized by irregularities in gonadotropin secretion, hyperandrogenism, chronic anovulation, and polycystic ovarian morphology. In addition, it is often associated with metabolic dysfunctions, most notably insulin resistance (IR). This disorder affects approximately 6–20% of individuals, primarily emerging during early adolescence, and considerably increases the risk of conditions such as impaired glucose tolerance, type 2 diabetes, endometrial cancer, cardiovascular diseases, dyslipidemia, and postpartum complications. To date, there is no standardized protocol for treating PCOS. Existing therapies primarily rely on personalized pharmacotherapy and lifestyle modifications. However, these treatments may often lead to adverse effects, and most medications prescribed for PCOS are used off-label and have not secured approval from the U.S. Food and Drug Administration specifically for this condition. Recently, natural compounds have garnered considerable attention due to their efficacy in hormone modulation and minimal toxicity. Substances such as myo-inositol, resveratrol, berberine, and quercetin have shown promise in mitigating PCOS symptoms. Their multi-target properties offer the potential to achieve outcomes unattainable by single-target pharmaceuticals, particularly in managing heterogeneous conditions. This review aims to comprehensively analyze *in vivo* and *in vitro* research alongside clinical interventions to evaluate the influence of natural compounds on the prevalence of PCOS and their therapeutic potential. These investigations lay the groundwork for developing innovative therapeutic strategies for PCOS.

## 1 Introduction

**Figure 1:**
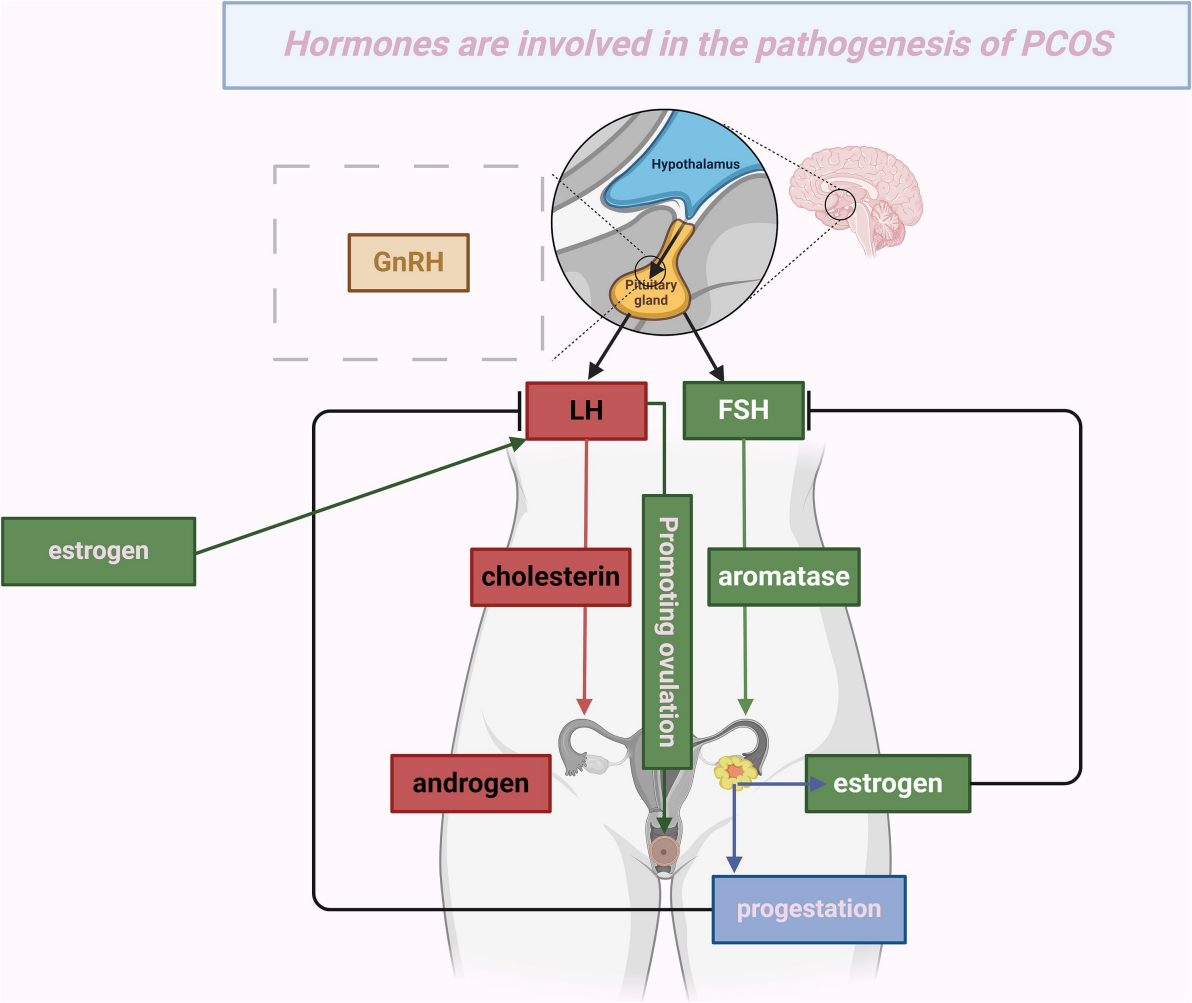
Hormones are involved in the pathogenesis of PCOS. This figure depicts the mechanism by which hypothalamic release of gonadotropin-releasing hormone (GnRH) activates the pituitary gland, stimulating the secretion of luteinizing hormone (LH) and follicle-stimulating hormone (FSH). LH facilitates the conversion of cholesterol into androgens in the ovaries. Concurrently, FSH, aided by the enzyme aromatase, promotes the conversion of androgens into estrogens. The resulting increase in estrogen levels causes a spike in LH, which triggers ovulation, showcasing the positive feedback loop between estrogen and LH. After ovulation, the corpus luteum produces progesterone and estrogen. These hormones exert negative feedback on LH and FSH, thereby regulating their levels. This cyclical process represents the normal human menstrual cycle. However, in conditions like polycystic ovary syndrome (PCOS), this hormonal regulation may become impaired, resulting in abnormal hormone levels.

PCOS is a heterogeneous endocrine disorder characterized by abnormal gonadotropin secretion, hyperandrogenism, persistent anovulation, and polycystic ovarian morphology. It is also associated with metabolic irregularities such as insulin resistance (IR) (1, 2). The prevalence of PCOS ranges from 6 to 20%, primarily manifesting during early adolescence (3). PCOS significantly elevates the risk of impaired glucose tolerance, type 2 diabetes (4), endometrial cancer (5), cardiovascular diseases, dyslipidemia (6), and postpartum complications (7). It also negatively impacts reproductive, metabolic, and psychological health (8). Research into the pathogenesis of PCOS remains limited (9), but current evidence highlights a notable association between hormonal fluctuations and PCOS pathogenesis, particularly in the levels of insulin, luteinizing hormone (LH), follicle-stimulating hormone (FSH), androgens, estrogens, and progesterone (10) (Figure 1).

To our knowledge, no unified standard for treating PCOS currently exists (11, 12). Since it cannot be completely cured, clinical treatment focuses on individualized drug therapy targeting specific symptoms, particularly hormonal medications. However, side effects are relatively common. For instance, anti-androgen medications reduce the biological effects of androgens through various mechanisms, thereby improving hirsutism and restoring ovulation (13, 14), but they may have potential hepatotoxicity (15). Combined oral contraceptives, which contain progestins and/or estrogens, are the most widely used drugs for PCOS (16). They regulate the menstrual cycle, prevent endometrial hyperplasia, and alleviate hyperandrogenic symptoms. However, long-term use raises the risks of IR, venous thrombosis (17), and menopausal concerns (18). Anti-estrogen medications, such as clomiphene citrate (CC), are first-line treatments for anovulatory infertility (19), but they can overstimulate the ovaries, leading to multiple pregnancies (20). Metformin significantly improves IR and hyperandrogenism, and its combination with CC enhances its ovulation-inducing effect (21, 22). Studies have also found that metformin positively affects the offspring of PCOS-IR rats (23). However, although thiazolidinediones, which are also insulin sensitizers, may cause adverse effects such as fluid retention and weight gain, their use is not recommended (19). Spironolactone improves hirsutism (24) but may cause intermenstrual bleeding (25). In addition, most of these drugs are used off-label and have not been approved by the U.S. Food and Drug Administration (FDA) for PCOS treatment (26).

Despite challenges in PCOS targeted therapy, natural products are gaining attention for their broad therapeutic potential (27). These compounds notably affect hormone regulation, and, being primarily derived from natural sources such as plants, animals, or microbes, they generally exhibit favorable safety profiles with low toxicity. Moreover, most natural compounds have multifaceted properties, allowing them to achieve outcomes that single-target medications cannot, especially in heterogeneous diseases. They can serve as complementary therapies, improving efficacy, reducing side effects, and decreasing reliance on prescription drugs when combined with traditional treatments (28–30). Several reviews have examined the effects of natural compounds on PCOS, but they often lack an in-depth analysis of hormone regulatory mechanisms or focus primarily on herbal extracts. This review aims to fill this gap. We focus on single-component natural compounds with well-defined mechanisms, such as terpenes, polyols, phenolics, and flavonoids. By reviewing preclinical and clinical studies, we explore their hormonal effects and therapeutic potential in PCOS, thereby providing a more reliable basis for clinical treatment.

## 2 Methods

This review collected studies on the effects of natural compounds on hormonal regulation in PCOS by searching the PubMed and PubMed Central databases. The search terms included, but were not limited to, “Polycystic Ovary Syndrome,” “PCOS,” “hormonal regulation,” “natural compounds,” “natural products,” “insulin resistance,” and “hyperandrogenism.” All collected articles and their references were reviewed, with a focus on natural compounds with well-defined chemical structures. Studies were excluded if they were not peer-reviewed, not in English, unrelated to hormonal regulation, or involved complex multi-component formulations.

## 3 Natural compounds in PCOS management

**Figure 2:**
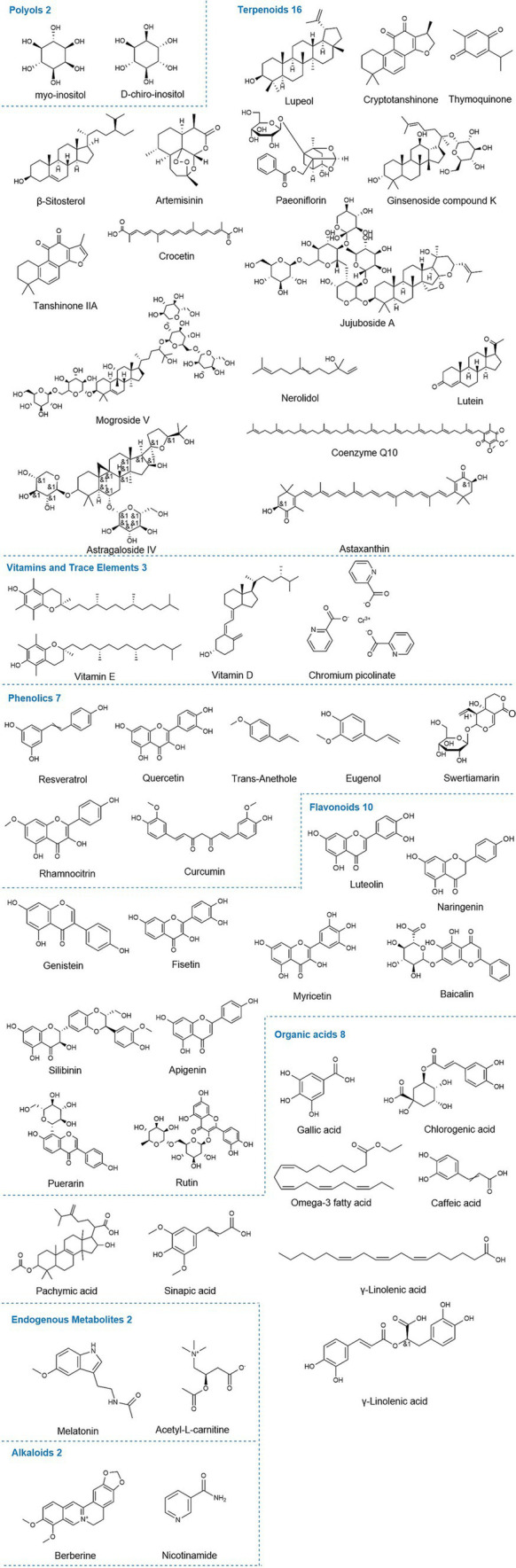
Chemical structures of relevant natural compounds. The figure illustrates the chemical structures of all individual compounds discussed in the article that can affect hormone levels in PCOS, categorized by polyols, terpenoids, endogenous metabolites, alkaloids, phenolics, flavonoids, organic acids, vitamins, and trace elements.

This study primarily involves the following categories of natural compounds (Figure 2). Polyols are organic compounds with multiple hydroxyl groups (-OH) directly attached to saturated carbon atoms and are widely found in nature. Terpenoids are classified based on the number of isoprene units, including monoterpenes, diterpenes, triterpenes, tetraterpenes, and sesquiterpenes. With over 40,000 known types, they are the largest class of plant metabolites, widely used in food, pharmaceuticals, chemicals, and biofuel development, with properties including antimalarial, anticancer, and hypoglycemic effects (31). Polysaccharides, complex macromolecules, are major components of traditional Chinese medicine, such as Astragalus, Ginseng, and Goji berries. Owing to their broad biological activities, they attract significant attention in research (32). Flavonoids, a subclass of phenolics, consist of two aromatic carbon rings connected by a three-carbon bridge. Both flavonoids and phenolics share several biological activities, such as antioxidant, anti-inflammatory, antimicrobial, anticancer, cardioprotective, and immune-boosting effects, which contribute significantly to disease prevention and treatment (33, 34). Alkaloids are organic compounds containing nitrogen atoms and exhibit potent antibacterial, antifungal, and antiviral activities. Although some are toxic, they also have significant therapeutic potential (35). Organic acids are compounds containing carboxyl groups (-COOH), which typically exhibit acidic reactions and may also contain other functional groups. Based on existing literature, other related compounds are classified as endogenous metabolites, vitamins, and trace elements.

## 4 Insulin in the pathogenesis of PCOS

Insulin is a primary anabolic hormone produced by pancreatic *β*-cells. It plays a crucial role in regulating the metabolism of glucose, lipids, and proteins. Its main function is to store energy when energy intake exceeds energy expenditure (36, 37). Under physiological conditions, insulin stimulates tissues such as the liver and adipose tissue to uptake glucose, helping to maintain glucose homeostasis. However, pathological conditions can lead to reduced insulin signaling and/or IR, resulting in decreased glucose transport. This may cause compensatory insulin secretion to sustain glucose homeostasis, which leads to hyperinsulinemia (38). IR refers to the decreased responsiveness of insulin-target tissues to physiological levels of insulin, which is a common feature of PCOS (39), although it is not included in the diagnostic criteria for PCOS. IR, along with resultant hyperinsulinemia, plays a significant role in synthesizing androgens beyond normal levels, primarily through two mechanisms. First, insulin enhances the responsiveness of human granulosa cells to LH (40). Elevated insulin levels can amplify LH stimulation, increasing androgen secretion and causing hyperandrogenemia (41, 42). Second, sex hormone-binding globulin (SHBG) acts as a transport protein for sex steroids. High insulin levels can inhibit serum SHBG levels, raising the concentration of free bioactive testosterone, ultimately leading to hyperandrogenemia (43, 44). Therefore, women with PCOS often have lower serum SHBG concentrations. In summary, insulin plays a critical role in the pathophysiology of PCOS.

### 4.1 The effect of natural compounds on insulin in PCOS models

#### 4.1.1 Polyols

Artini et al. (45) conducted folic acid therapy in patients with PCOS and found that the combination of myo-inositol (MYO) and folic acid significantly reduced serum insulin levels, thereby improving oocyte quality and pregnancy rates. Another randomized double-blind trial with a similar methodology confirmed these results (46). Donà et al. (47) examined erythrocytes in patients with PCOS before and after treatment. The results indicated that MYO may lower fasting serum insulin (FINS) levels through the insulin-related metabolic pathway, thereby improving IR-related hyperinsulinemia. Earlier studies also suggested that D-chiro-inositol (DCI) supplementation could lower serum insulin and enhance insulin sensitivity in PCOS patients with impaired glucose tolerance, thus improving ovulatory function (48).

#### 4.1.2 Terpenoids

Research has indicated that lupeol treatment could downregulate the expression of TLR-4 and TLR-2 genes induced by DHEA in PCOS mice. This effect may arise from its antioxidant and anti-inflammatory properties, which result in reduced insulin levels in PCOS mice (49). A randomized double-blind placebo-controlled trial demonstrated that 12 weeks of coenzyme Q10 supplementation can lower serum insulin levels in patients with PCOS (50). Wen et al. (51) reported that astragaloside IV can reduce serum insulin levels in DHEA-induced PCOS rats in a dose-dependent manner, thereby improving IR. Furthermore, astaxanthin significantly reduced insulin levels in infertile PCOS patients and regulated glucose and lipid metabolism (52).

#### 4.1.3 Polysaccharides

Hu et al. (53) discovered that Dendrobium nobile-derived polysaccharides lower insulin levels in PCOS rats induced by letrozole and a high-fat diet by activating SIRT2, which subsequently improves IR and restores glycolytic pathways. Zhou et al. (54) found that in a letrozole-induced PCOS model, *Irpex lacteus* polysaccharides reduced insulin levels by enhancing antioxidant enzyme expression, thereby regulating glucose and lipid metabolism. Studies have also indicated that trehalose may lower insulin levels in DHEA-induced PCOS mice by downregulating the ACE/AngII/AT1R pathway, thus alleviating related symptoms (55).

#### 4.1.4 Alkaloids

An et al. (56) executed a randomized controlled trial (RCT) involving patients with PCOS preparing for IVF treatment. They found that initial treatment with berberine induced a significant decrease in FINS levels among these patients, thus enhancing pregnancy outcomes. Similarly, Li et al. (57) observed that in a DHEA-induced PCOS rat model, berberine effectively lowered FINS levels, showing superior efficacy in IR management compared with metformin. Furthermore, Yu et al. (58) proposed that berberine’s mechanism for reducing serum insulin levels might involve the PI3K/AKT signaling pathway.

#### 4.1.5 Phenolics

Phenolics have garnered considerable attention in recent studies. Liang et al. (59) demonstrated that resveratrol modulates SIRT2 to lower serum insulin levels in a letrozole- and high-fat diet-induced rat model of PCOS, thereby alleviating ovarian damage in these rats. Similarly, Shah et al. (60) found that quercetin reduces serum insulin levels in letrozole-induced PCOS mice models, possibly linked to quercetin’s ability to decrease plasma vascular endothelial growth factor levels. Rezvan et al. (61) reported that quercetin may lower serum insulin levels in patients with PCOS by increasing adiponectin concentrations, which improves IR. In addition, research has shown that trans-anethole treatment can reduce insulin levels in testosterone-induced PCOS rat models through its antioxidant properties and protective effects on liver and kidney tissues (62).

#### 4.1.6 Flavonoids

Jamilian et al. (63) conducted a randomized, double-blind, placebo-controlled trial whose findings indicated that prolonged administration of soy isoflavones for 12 weeks diminished serum insulin levels in individuals with PCOS, thereby enhancing their insulin sensitivity. Zhou et al. (64) reported that total flavonoids sourced from *Nervilia fordii* lowered serum insulin levels in DHEA-induced SD rat PCOS models. This effect may correlate with the modulation of the JAK2/STAT3 signaling pathway primarily influenced by IL-6. Furthermore, genistein substantially decreased serum insulin levels in estradiol valerate (EV)-induced Wistar rat models, consequently improving IR (65). In the subsequent two studies, the therapeutic benefits of genistein on insulin were reaffirmed (66, 67). Huang and Zhang (68) revealed that luteolin attenuated FINS concentrations in letrozole-induced and high-fat diet-induced PCOS rats by modulating the PI3K/AKT signaling pathway, thereby improving IR. In addition, fisetin has the potential to lower insulin levels in letrozole-induced Wistar rats PCOS models by enhancing the expression of SIRT1 and AMPK in the ovaries, thus regulating glucose homeostasis (69). Wu et al. (70) reported that in letrozole-induced PCOS SD rats, naringenin treatment reduced FINS levels and improved IR. The potential mechanism may involve the SIRT1/PGC-1α signaling pathway and gut microbiota.

#### 4.1.7 Organic acids

Shah et al. (71) demonstrated that gallic acid administration can decrease serum insulin levels in letrozole-induced PCOS mice. This effect may stem from the enhanced ovarian antioxidant capacity linked to gallic acid. In addition, another study identified that chlorogenic acid potentially lowers insulin levels in letrozole-induced PCOS mice by modulating adiponectin levels and improving antioxidant capacity (72). Mohammadi et al. (73) showed that omega-3 fatty acid supplementation can diminish serum insulin levels in patients with PCOS by increasing adiponectin levels, thereby reducing the incidence of PCOS-associated complications. Research findings also indicate that caffeic acid may decrease FINS levels in PCOS rats by mitigating endoplasmic reticulum (ER) stress and oxidative stress, thus enhancing their insulin sensitivity (74). Further investigations revealed that pachymic acid can lower serum insulin levels in DHEA-induced PCOS mice by regulating the CYP-17, IRS-1, and GLUT4 protein expression, thereby effectively improving IR within the ovarian tissues of these mice (75).

#### 4.1.8 Endogenous metabolites

A randomized, double-blind, placebo-controlled clinical trial revealed that a 12-week melatonin regimen significantly reduced serum insulin levels in PCOS patients, aiding glucose homeostasis. This effect is likely linked to melatonin’s ability to upregulate the expression of the peroxisome proliferator-activated receptor gamma (PPAR-γ) gene (76). In another RCT, Tauqir et al. (77) incorporated acetyl-L-carnitine into a treatment protocol with metformin and pioglitazone. After 12 weeks, the combination therapy exhibited markedly improved efficacy compared to the control group, confirming acetyl-L-carnitine’s role in lowering FINS levels and its synergistic effect with other pharmacotherapies.

#### 4.1.9 Vitamins and trace elements

Research indicates that vitamin D supplementation effectively reduces serum insulin levels induced by EV in PCOS rat models and enhances their ovarian tissue protective mechanisms (78). In addition, Ashoush et al. (79) conducted an RCT with 100 patients with PCOS, who received 1,000 μg chromium picolinate (CrP) for 6 months. The results demonstrated that CrP lowered FINS levels, improved IR, enhanced ovulation, and regulated menstrual cycles, showcasing its effective therapeutic role in addressing PCOS.

Although insulin is not a reproductive hormone, it plays a crucial role in the multi-stage, comprehensive management of PCOS. It serves as an important link between PCOS and various related diseases. Therefore, studying the effects of natural compounds on insulin in PCOS is of significant interdisciplinary importance. Overall, flavonoids play a prominent role in regulating insulin levels in PCOS. The treatment approach involves modulating various signaling pathways such as JAK2/STAT3, PI3K/AKT, and SIRT1/PGC-1α, boosting SIRT1 and AMPK expression, and regulating gut microbiota. Organic acids and phenolics have also received considerable attention. However, research on alkaloids has primarily focused on berberine, especially the PI3K/AKT pathway. Moreover, other compounds also enhance insulin sensitivity through various mechanisms, such as modulating ACE/AngII/AT1R, TLR-4, and TLR-2. These interventions not only improve ovulation function but also reduce the risk of diabetes, cardiovascular disease, and other PCOS-related complications.

## 5 LH in the pathogenesis of PCOS

LH is secreted in a pulsatile pattern by gonadotrope cells in the pituitary gland. This secretion is directly stimulated by gonadotropin-releasing hormone (GnRH) produced in the hypothalamus (80). LH is vital for follicular growth and maturation. In general, researchers often use LH pulse frequency to indirectly assess GnRH pulse activity. Under normal physiological conditions, LH promotes androgen production during the follicular phase, thereby facilitating estrogen synthesis and oocyte maturation. At the midpoint of the menstrual cycle, LH levels increase sharply, playing a crucial role in triggering ovulation. During the luteal phase, LH aids in progesterone secretion, although its concentration typically declines (81). In pathological contexts, excessive LH levels can inhibit follicular development (82). In patients diagnosed with PCOS, both the frequency and amplitude of GnRH pulses increase, selectively increasing LH levels. This leads to a significant increase in LH synthesis and secretion. The increased ovarian androgen production leads to hyperandrogenemia (83–85). In addition, the absence of an LH surge is a key characteristic of PCOS (10). Therefore, LH is crucial for understanding the pathophysiology of PCOS.

### 5.1 The effect of natural compounds on LH in PCOS models

#### 5.1.1 Polyols

Artini et al. (45) found that folic acid, as a baseline treatment for patients with PCOS, significantly reduced LH levels after 12 weeks of combined treatment with MYO, helping to normalize the menstrual cycle. This suggests the effectiveness of MYO in lowering LH levels.

#### 5.1.2 Terpenoids

Terpenoids have received considerable attention for their potential impact on PCOS. The study highlights that *β*-sitosterol may enhance gut microbiota balance in DHEA-induced PCOS mice, thereby lowering their LH levels (86). Yang et al. (87) established a PCOS rat model utilizing daily injections of human chorionic gonadotropin (HCG) and insulin (Ins) over 22 days. Their results indicated that cryptotanshinone effectively reduced LH levels in PCOS rats by influencing the HMGB1/TLR4/NF-κB signaling cascade, thereby alleviating reproductive dysfunction. A study revealed thymoquinone’s antioxidant and anti-apoptotic effects, which contributed to decreased LH levels in letrozole-induced PCOS rat models, thereby mitigating follicular atresia (88). In addition, paeoniflorin (89) and astragaloside IV (51) lowered serum LH concentrations in DHEA-induced PCOS rats by modulating the TGF-β1/Smads and PPARγ signaling pathway, promoting ovarian health and regulating the reproductive cycle. Rezaei-Golmisheh et al. (90) reported that lupeol attenuates LH levels in DHEA-induced PCOS mice models through its antioxidant capacity, thereby improving fertility outcomes. Other investigations demonstrated that crocetin lowers serum LH concentrations in DHT-induced PCOS mice by modulating kisspeptin neuron activity, which supports follicular maturation and reduces ovulatory issues (91). Finally, research findings indicate that ginsenoside compound K lowers LH levels in DHEA-induced PCOS rats by modulating CXCL14 expression in brown adipose tissue, thus restoring the estrous cycle (92). Türkmen et al. (93) further established that nerolidol decreases LH concentrations in DHEA-induced PCOS SD rats, addressing hormone secretion irregularities.

#### 5.1.3 Polysaccharides

The polysaccharide extracted from *Irpex lacteus* lowers LH levels in letrozole-induced PCOS rat models, improving hormonal balance, likely due to its antioxidant effects and suppression of the TGF-β1/Smad signaling pathway (54).

#### 5.1.4 Phenolics

Phenolics attract considerable research interest. Chen et al. (94) exposed neonatal SD rats to tributyltin to induce PCOS. This exposure altered LH concentrations, disrupted estrous cycles, and inhibited follicular development. Resveratrol reversed these effects by facilitating calcium ion transport and activating CaMKIIβ, thus facilitating the repair of transzonal projections. Shah and Patel (95) showed that quercetin could reduce LH levels in propionate testosterone-induced PCOS rats by inhibiting PI3K levels and CYP17A1 gene expression, thereby regulating ovarian steroidogenesis. Shah et al. (96) created a PCOS model in adult Swiss Albino mice using letrozole and identified that curcumin could lower LH levels by modifying the androgen-adiponectin balance in circulation, thus preventing ovarian dysfunction. Subsequently, Zhang et al. (97) discovered that curcumin’s ability to reduce LH levels in DHEA-induced PCOS rats might relate to inhibiting the ER stress-related IRE1α-XBP1 pathway alongside activating the PI3K/AKT signaling pathway. Zhou et al. (98) noted that rhamnocitrin could decrease LH levels in letrozole-induced PCOS SD rats, facilitating the recovery of ovarian tissue, possibly through enhanced PPAR-*γ* activity and the inhibition of the TGF-β1/Smad pathway. Furthermore, evidence suggests that eugenol might lower serum LH levels in EV-induced PCOS model Wistar rats by modulating COX-2 and PPAR-α gene expression, thereby ameliorating ovarian cysts (99).

#### 5.1.5 Flavonoids

Flavonoids exhibit notable effects in PCOS models. Total flavonoids have been shown to reduce LH levels in DHEA-induced PCOS rats, restoring ovulation. This effect may be associated with modulation via the IL-6-mediated JAK2/STAT3 signaling pathway (64). Khezri et al. (65) found similar effects with genistein in EV-induced PCOS rats. In addition, silybin can lower LH levels in letrozole-induced PCOS rats through its antioxidant and anti-inflammatory actions, normalizing reproductive cycles and improving ovarian and uterine morphology (100). Baicalin may decrease LH levels in DHEA-induced PCOS rats by regulating miR-874-3p/FOXO3 and miR-144/FOXO1 expression in ovarian tissue (101). Luteolin has also shown potential to reduce LH levels in letrozole- and high-fat diet-induced PCOS rats, possibly through Nrf2 activation and antioxidant effects (68). Zheng et al. (102) propose that myricetin may lower LH levels in DHEA-induced PCOS mice by activating brown adipose tissue (BAT), improving ovarian function and metabolic irregularities. Finally, Wu et al. (70) showed that naringenin significantly reduced LH levels and improved ovarian function in letrozole-induced PCOS SD rats.

#### 5.1.6 Organic acids

Research reveals that omega-3 polyunsaturated fatty acids (PUFAs) can lower LH levels in DHEA-induced PCOS mice. This modulation may influence ovarian androgen production and its conversion (103). Lan et al. (104) found that sinapic acid decreases LH levels in letrozole-induced PCOS rats, aiding in regulating ovulation and alleviating ovarian fibrosis. In similar models, rosmarinic acid may reduce LH levels through its anti-inflammatory and anti-angiogenic effects (105). Furthermore, chlorogenic acid has been shown to reduce LH levels in letrozole-induced PCOS model mice, likely linked to its effect on adiponectin levels, antioxidant capacity, and anti-inflammatory properties (72). Khoshvaghti et al. (106) found that ellagic acid can diminish LH levels in EV-induced PCOS model rats, facilitating the recovery of follicular development.

#### 5.1.7 Endogenous metabolites

Tauqir et al. (77) found that acetyl-L-carnitine can lower LH levels in patients with PCOS, thereby restoring hormonal balance.

#### 5.1.8 Vitamins

Studies indicate that vitamin D supplementation can lower LH levels in rat models of PCOS caused by EV, thus safeguarding ovarian tissue (78).

**Figure 3:**
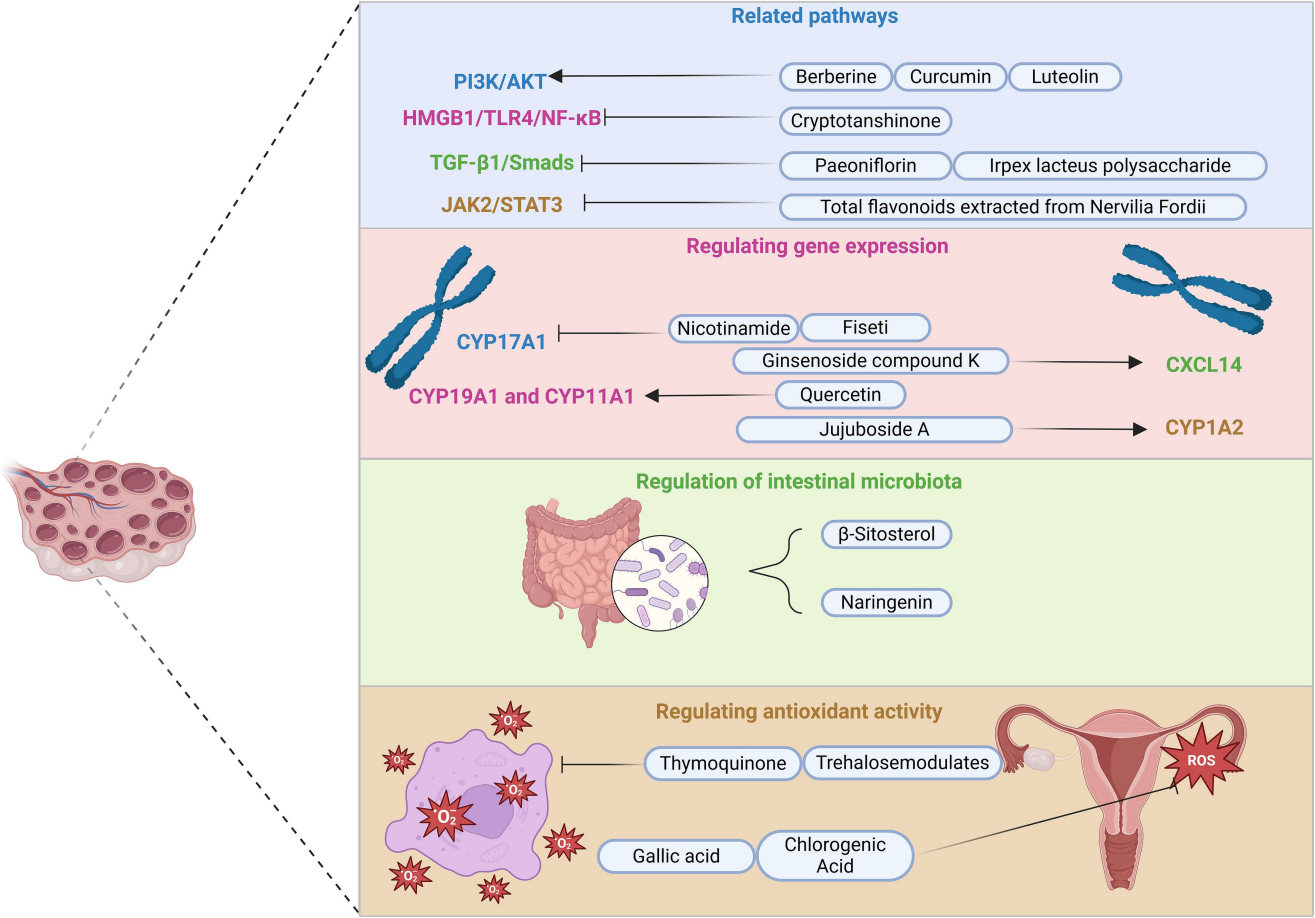
Main intervention pathways of natural compounds in PCOS. This figure illustrates four primary intervention pathways of natural compounds in PCOS management: related signaling pathways, regulating gene expression, regulation of intestinal microbiota, and antioxidant activity. In addition, it includes some compounds and their targets, with symbols such as activation and inhibition arrows used to describe their mechanisms of action. For instance, berberine, curcumin, and luteolin activate the PI3K/AKT pathway, whereas cryptotanshinone inhibits the HMGB1/TLR4/NF-κB pathway. Paeoniflorin and *Irpex lacteus* polysaccharide suppress the TGF-β1/Smads pathway, and total flavonoids derived from *Nervilia Fordii* inhibit JAK2/STAT3 signaling. In addition, nicotinamide downregulates CYP17A1 gene expression, while ginsenoside compound, jujuboside A, and quercetin specifically activate CXCL14, CYP1A2, CYP19A1, and CYP11A1. Furthermore, naringenin and β-sitosterol modulate gut microbiota, whereas trehalose and thymoquinone mitigate oxidative stress. Finally, gallic acid and chlorogenic acid enhance the antioxidant capacity of the ovaries. PI3K, phosphoinositide 3-kinase; AKT, protein kinase B; HMGB1, high mobility group box 1; TLR4, Toll-like receptor 4; NF-κB, nuclear factor kappa B; TGF-β1, transforming growth factor-beta 1; JAK2, Janus kinase 2; STAT3, signal transducer and activator of transcription 3; CYP17A1, cytochrome P450 family 17 subfamily A member 1; CXCL14, C-X-C motif chemokine ligand 14; CYP1A2, cytochrome P450 family 1 subfamily A member 2; CYP19A1, cytochrome P450 family 19 subfamily A member 1; CYP11A1, cytochrome P450 family 11 subfamily A member 1.

Research on regulating LH dysregulation in PCOS has predominantly focused on terpenoids, phenolics, and flavonoids due to their distinct chemical structures. These compounds have been shown to enhance gene expression modulation related to CXCL14, kisspeptin, CYP17A1, COX-2, and PPAR-α. In addition, several key signaling pathways, including HMGB1/TLR4/NF-κB, TGF-β1/Smads, IRE1α-XBP1, and PI3K/AKT/Nrf2, play a crucial role in regulating LH and metabolic homeostasis. In contrast, research on the regulatory effects of polysaccharides, polyols, vitamins, and other compounds on LH in PCOS is relatively limited, with only observational effects and insufficient investigation into the underlying molecular and cellular mechanisms (Figure 3).

## 6 FSH in the pathogenesis of PCOS

FSH is a heterodimeric glycoprotein produced in pulses by the gonadotropic cells of the anterior pituitary. Its secretion is regulated by the pulsatile release of GnRH from the hypothalamus, along with factors such as neuropeptides like kisspeptin, gonadal steroids, inhibin, and others. A slower frequency of GnRH pulses typically enhances the synthesis and release of FSH (82, 107). Under normal physiological conditions, FSH initiates follicular growth and, via aromatase activity, enables granulosa cells to convert androgens into estrogens (81). Once a dominant follicle appears, FSH levels drop, causing the atresia of other follicles and halting their development (108). The dominant follicle thus becomes the sole candidate for ovulation. In patients with PCOS, disrupted neuroendocrine regulation leads to increased frequency and amplitude of GnRH pulses, while FSH secretion remains relatively suppressed at low normal levels (83, 85). This disruption results in impaired follicular development, potentially caused by pituitary desensitization from heightened GnRH stimulation (109). In summary, FSH plays a vital role in the pathophysiology of PCOS.

### 6.1 The effect of natural compounds on FSH in PCOS models

#### 6.1.1 Polyols

For patients undergoing ovulation induction and intrauterine insemination, MYO supplementation can reduce the dosage of rFSH administered, resulting in favorable clinical outcomes (110).

#### 6.1.2 Terpenoids

Terpene compounds have shown promise in recent studies. Research indicates that *β*-sitosterol can elevate FSH levels in DHEA-induced PCOS model mice, likely due to its modulatory effects on gut microbiota (86). Alaee et al. (88) found that thymoquinone increases the transcription level of the *GPx1* gene while decreasing *Bax* gene expression and the *Bax/Bcl2* ratio. This occurs through the stimulation of the antioxidant system and the inhibition of apoptotic pathways, ultimately enhancing serum FSH levels in letrozole-induced PCOS model rats, thereby promoting follicular development. Findings have also revealed that crocetin can elevate serum FSH levels in DHT-induced PCOS model mice. This effect occurs by increasing AVPV-kisspeptin expression and reducing ARC-kisspeptin expression, thereby facilitating follicular development (91). In addition, Türkmen et al. (93) discovered that nerolidol can enhance FSH levels in DHEA-induced PCOS model SD rats, thereby regulating hormonal secretion disorders.

#### 6.1.3 Phenolics

Phenolics play a crucial role in PCOS management. A triple-blind RCT indicated that resveratrol may enhance FSH levels in patients with PCOS by lowering androgen-derived steroid concentrations (111). This process facilitates follicular maturation and improves oocyte quality, ultimately enhancing pregnancy outcomes. Shah et al. (96) established a PCOS model in adult Swiss Albino mice using letrozole. Their study revealed that curcumin, a turmeric extract, might boost FSH levels by exerting anti-hyperlipidemic and antioxidant effects, along with raising circulating adiponectin levels. Furthermore, Zhang et al. (97) demonstrated that curcumin’s therapeutic mechanism likely includes the inhibition of the IRE1*α*-XBP1 pathway associated with ER stress and the activation of the PI3K/AKT signaling pathway. Kokabiyan et al. developed a PCOS model in Wistar rats through continuous EV administration, discovering that eugenol could elevate serum FSH levels by modulating the expression of the COX-2 and PPAR-α genes, thereby enhancing follicular development (99). Zhou et al. (98) reported that rhamnocitrin increases FSH levels in letrozole-induced PCOS rats and improves ovarian morphology by acting on the HPG axis. In addition, quercetin has been shown to elevate FSH levels in mature Parkes strain mice with letrozole-induced PCOS, effectively reversing follicular degeneration and reinstating normal ovarian function (60).

#### 6.1.4 Flavonoids

Flavonoids have recently gained considerable interest in scientific research. Investigations reveal that genistein can boost FSH levels in PCOS model rats induced by EV, which leads to improved follicle development and maturation (65). Total flavonoids may elevate FSH levels in DHEA-induced PCOS model SD rat models by downregulating IL-6 expression, thereby supporting ovarian function recovery (64). Research conducted by Huang and Zhang (68) demonstrates that luteolin can increase FSH levels in PCOS model SD rats established with letrozole and a high-fat diet, which subsequently modulates the reproductive cycle and enhances ovarian morphology. Moreover, Wu et al. (70) reported that in letrozole-induced PCOS rats, naringenin caused hormonal changes similar to those caused by luteolin.

#### 6.1.5 Organic acids

Shah et al. established PCOS mouse models using letrozole. Two separate studies found that gallic acid (71) and chlorogenic acid (72) both increased FSH levels in PCOS mice by regulating the expression of adiponectin and its receptor, thereby promoting follicular development. In addition, sinapic acid (SA) was shown to raise letrozole-induced FSH levels in PCOS model SD rats, influencing ovulation and alleviating ovarian fibrosis (104).

#### 6.1.6 Vitamins

Two subsequent studies indicate that vitamin D supplementation may increase FSH levels in EV-induced PCOS rats, which in turn facilitates follicular generation and growth (78, 112).

Various natural compounds have demonstrated promising therapeutic effects in both preclinical and clinical models, promoting follicle maturation, restoring ovulatory function, and improving fertility outcomes by enhancing FSH levels. These compounds regulate various therapeutic mechanisms, from gut microbiota modulation to exerting antioxidant effects, highlighting the complexity of PCOS treatment and the need for multi-target strategies. With the widespread use of rFSH technology, understanding how these compounds interact with it could lead to more effective treatment options for PCOS patients.

## 7 Androgens in the pathogenesis of PCOS

Androgens encompass hormones such as dehydroepiandrosterone sulfate (DHEAS), dehydroepiandrosterone (DHEA), androstenedione (A4), testosterone (T), and dihydrotestosterone (DHT). These hormones mainly originate from the adrenal glands and ovaries, where they are derived from cholesterol through enzymatic processes triggered by LH. As precursors of estradiol, androgens are vital for the normal functioning of ovarian physiology (113, 114). While many aspects of PCOS remain unclear, it is widely accepted that elevated androgen levels significantly contribute to the reproductive and metabolic complications associated with PCOS. Notably, in a large cohort of clinically hyperandrogenic individuals, 72.1% of females were diagnosed a PCOS diagnosis (115). Hyperandrogenemia is considered a principal clinical characteristic of PCOS (116). Furthermore, elevated androgen levels can impair hypothalamic sensitivity to progesterone and estradiol, thereby disrupting the inhibitory regulation of GnRH pulsatility. This disruption establishes a detrimental feedback loop between hyperandrogenemia and hypothalamic–pituitary–ovarian axis dysfunction (85). Increased levels of LH and insulin further stimulate androgen secretion, undermining ovarian function. In addition, excessive androgen production triggers lipolysis, raising free fatty acid levels, altering muscle tissue composition and performance, and leading to IR and hyperinsulinemia. Consequently, this creates a vicious cycle among IR, hyperinsulinemia, and hyperandrogenemia in patients with PCOS (117–119). In conclusion, androgens are essential in the pathophysiology of PCOS.

### 7.1 The effect of natural compounds on androgens in PCOS models

#### 7.1.1 Polyols

Polyols play an important role in medical research. Artini et al. (45) found that a combination therapy of MYO and folic acid is more effective than folic acid alone in treating PCOS, significantly lowering serum testosterone levels and alleviating symptoms. Donà et al. (47) analyzed erythrocytes in patients with PCOS before and after treatment, observing that MYO substantially reduced serum testosterone and androstenedione levels through the phosphoinositide-related signaling pathway, thereby alleviating systemic inflammation. Furthermore, studies indicate that DCI supplementation can diminish serum-free testosterone levels in patients with PCOS, thereby supporting ovulation (48). MYO and DCI maintain a critical balance within the human body. In PCOS-related studies, the optimal MYO/DCI ratio is suggested to be 40:1 (120). Consequently, Fedeli et al. (121) found that at this ratio, MYO and DCI reduced serum DHEA levels in PCOS mice by modulating androgen enzyme expression and increasing CYP19A1 and FSHR synthesis.

#### 7.1.2 Terpenoids

Terpenoids play a crucial role in various biological processes. Liu et al. (122) reported in *Science* that artemisinins inhibit the activity of cytochrome P450 family 11 subfamily A member 1 (CYP11A1) by directly interacting with lon peptidase 1 (LONP1). This inhibition results in reduced androgen synthesis in a DHEA-induced PCOS rat model, thereby enhancing fertility. Meanwhile, Malekinejad et al. (49) found that lupeol may regulate *TLR-4* and *TLR-2* gene expression and serum TNF-*α* levels in DHEA-induced PCOS mice, thereby reducing serum testosterone levels and treating hyperandrogenemia. A subsequent study indicated that its therapeutic effects are also associated with a reduction in oxidative stress biomarkers (90). Using the same inducer, Ye et al. (92) found that ginsenoside compound K also reduces serum testosterone levels in PCOS rats by stimulating CXCL14 expression in BAT. In similar models, nerolidol (93) improved reproductive endocrine function through its antioxidant effects, whereas astragaloside IV (51) exerted a comparable effect by enhancing autophagy. Huang et al. (123) established a PCOS model in Sprague Dawley rats by gavaging them with letrozole and a high-fat diet. They found that mogroside V treatment may reduce serum testosterone levels and promote follicular development and ovulation by upregulating the expression of LDHA, HK2, and PKM2. Another study found that crocetin can decrease serum testosterone levels in DHT-induced PCOS mice by restoring kisspeptin neurons (91). Yang et al. (87) demonstrated that cryptotanshinone lowers testosterone levels in HCG and Ins-induced PCOS rat models by inhibiting the HMGB1/TLR4/NF-κB signaling pathway, ultimately improving reproductive function. In addition, tanshinone IIA, also derived from Danshen, produces similar positive outcomes in estradiol-induced PCOS mice by regulating FSHR and aromatase expression (124).

#### 7.1.3 Polysaccharides

Zhou et al. (54) found that the *Irpex lacteus* polysaccharide may decrease serum testosterone levels in letrozole-induced PCOS rats. This effect could reduce fat accumulation and improve ovarian fibrosis, possibly through inhibition of the TGF-β1/Smad pathway. Gao et al. (55) induced a PCOS model using a high-fat diet and DHEA. Their research indicated that trehalose supplementation can reduce serum testosterone levels in PCOS model mice, thereby alleviating the associated symptoms. This beneficial effect likely stems from trehalose’s capability to alleviate oxidative stress and cell death in ovarian granulosa cells.

#### 7.1.4 Alkaloids

An et al. (56) conducted an RCT to assess IVF preparation in patients with PCOS. The study showed that a 3-month pre-treatment with berberine significantly reduced serum total testosterone levels, thereby improving pregnancy outcomes. Shen et al. (125) further studied DHEA-induced PCOS rats and suggested that berberine’s regulatory mechanism may involve cell apoptosis and the regulation of key signaling molecules, such as TLR4, LYN, PI3K, AKT, NF-κB, TNF-*α*, IL-1, and IL-6, and caspase-3 expression. In addition, the study indicated that nicotinamide could decrease serum testosterone levels in letrozole-induced PCOS rats by downregulating the gene expression of CYP17A1, thereby contributing to the regulation of the estrous cycle (126).

#### 7.1.5 Phenolics

In a triple-blind RCT, researchers observed that resveratrol treatment significantly decreased serum testosterone levels in patients with PCOS. This decrease influenced the expression of the *VEGF* and *HIF1* genes within the angiogenic pathways of granulosa cells, thereby enhancing pregnancy outcomes (111). Shah et al. (60) proposed that quercetin reduces serum testosterone levels in letrozole-induced PCOS mice by upregulating CYP19a1 and CYP11a1, thus restoring normal ovarian function. Similarly, Shah and Patel (95) suggested that quercetin affects testosterone levels by suppressing the gene expression of CYP17A1 via the inhibition of the PI3K pathway. Research has demonstrated that curcumin normalizes serum testosterone levels in letrozole-induced PCOS rats (127). In a 12-week clinical study, curcumin also demonstrated its effectiveness in alleviating hyperandrogenism in patients with PCOS (128). Subsequently, Zhang et al. (97) reported that the effect of curcumin may be related to the inhibition of the ER stress-related IRE1*α*-XBP1 pathway and the activation of the PI3K/AKT signaling pathway. Zhou et al. (98) identified that rhamnocitrin may decrease serum testosterone levels in letrozole-induced PCOS model SD rats by reducing malondialdehyde (MDA) production and increasing serum superoxide dismutase (SOD) activity, resulting in fewer cysts and improved ovarian morphology. Eugenol might lower serum testosterone levels in letrozole-induced PCOS model Wistar rats through the modulation of the COX-2 and PPAR-α gene expressions, thereby affecting glucose and lipid metabolism (99). Furthermore, Moradi Negahdari et al. (129) reported that trans-anethole could dose-dependently reduce serum testosterone and DHEAS levels in testosterone-induced PCOS model rat models, exhibiting anti-androgenic effects comparable to those of metformin.

#### 7.1.6 Flavonoids

Flavonoids demonstrate considerable effects in the treatment of PCOS. Jamilian et al. (63) found that soy isoflavone supplementation lowers serum total testosterone levels in patients with PCOS, which assists in managing the condition. Research highlights that baicalin substantially reduces free testosterone levels in DHEA-induced PCOS rat models. This mechanism is linked to the miR-874-3p/FOXO3 and miR-144/FOXO1 pathways within ovarian tissues (101). Total flavonoids can produce similar positive effects by regulating the IL-6-mediated JAK2/STAT3 signaling cascade (64).

In addition, research conducted by Peng et al. (130) on DHEA-induced PCOS rat models demonstrated that apigenin effectively reduces serum testosterone levels, likely due to its antioxidant effects and the suppression of inflammatory cytokine expression. Similarly, silibinin also reduces testosterone levels in PCOS rats induced by letrozole through its antioxidant and anti-inflammatory properties (100). Li et al. (131) treated all patients with PCOS using a combination of Diane-35 and metformin, discovering that those in the puerarin subgroup markedly decreased their serum T levels by increasing SHBG and SOD levels in circulation, thus achieving a positive impact on hyperandrogenemia management. Moreover, luteolin was effective in normalizing estrous cycles and improving ovarian morphology while reducing serum T levels in letrozole- and high-fat diet-induced PCOS SD rat models (68). Fisetin, rutin, and naringenin all alleviate hyperandrogenism in letrozole-induced PCOS rats. Their mechanisms involve fisetin reducing CYP17A1 expression (69), rutin exerting antioxidant effects (132), and naringenin modulating the gut microbiota and the SIRT1/PGC-1α pathway (70).

#### 7.1.7 Organic acids

Chlorogenic acid can decrease serum testosterone levels in letrozole-induced PCOS model mice, likely due to its regulation of adiponectin levels, antioxidant capacity, and anti-inflammatory effects (72). Shah et al. (71) developed a PCOS model by administering letrozole via gavage to adult Parkes strain mice for 21 days. They found that gallic acid may lower serum testosterone levels in PCOS model mice by increasing the mRNA expression of CYP11a1 and CYP19a1. Research indicates that SA can enhance the serum activity of endogenous antioxidants and reduce malondialdehyde (MDA) production, thereby lowering serum testosterone levels in letrozole-induced PCOS model rats, promoting follicular development and improving atresia (104). A previous study showed that omega-3 polyunsaturated fatty acids can reduce serum testosterone levels in DHEA-injected PCOS model mice, potentially related to their anti-inflammatory effects on the ovaries (103). Similarly, Chiang et al. (74) found that caffeic acid reduces androstenedione and testosterone levels in DHEA-induced PCOS rats by modulating protein expression in steroid hormone synthesis and ER stress. Prabhu et al. (133) reported that administering *γ*-linolenic acid at a dose of 50 mg/kg can reduce serum and ovarian DHEA concentrations in DHEA-induced PCOS model rats, alleviating inflammation and improving tissue structure.

#### 7.1.8 Vitamins

Vitamin D supplementation has been shown to decrease testosterone levels in EV-induced PCOS model rats, protecting ovarian tissues from PCOS-related damage (78). Izadi et al. observed that vitamin E can lower serum total testosterone levels in patients with PCOS, providing adjunctive treatment for the condition (134).

#### 7.1.9 Endogenous metabolites

A study suggests that melatonin can exert effects comparable to metformin, significantly lowering testosterone levels in letrozole-induced PCOS mice, thereby reducing uterine volume (135). Basheer et al. (136) investigated melatonin’s effects, revealing it decreases serum testosterone levels in letrozole-induced PCOS model rats through the modulation of steroidogenic enzyme activity. Yu et al. (137) further identified that melatonin also reduces serum testosterone in patients with PCOS via the ERK pathway, which aids in oocyte development.

As one of the key hormones in PCOS, elevated androgen levels directly contribute to several typical clinical manifestations, including hirsutism, acne, and seborrheic dermatitis, primarily affecting appearance and quality of life, especially in younger patients. Early intervention using effective natural compounds may help alleviate these symptoms, thereby reducing the reliance on medication. Current research shows that androgen regulation in PCOS has been the most extensively studied, with various treatment mechanisms identified, including the upregulation of LDHA, HK2, and PKM2 expression, as well as the modulation of pathways such as IRE1α-XBP1 and ERK. In addition, signaling pathways such as TGF-β1/Smads and SIRT1/PGC-1α could regulate multiple hormones. Further investigation into these mechanisms may reveal new therapeutic options for PCOS management.

## 8 Progesterone in the pathogenesis of PCOS

Progesterone plays a vital role in the pathogenesis and treatment of PCOS. This steroid hormone primarily originates from the ovarian corpus luteum, with smaller contributions from the adrenal glands and placental synthesis during pregnancy (138, 139). It facilitates several reproductive functions in women, including oocyte maturation, ovulation, menstruation, pregnancy, and mammary development (85). In addition, it plays an antagonistic role against androgens and estrogens. Under normal physiological circumstances, the cyclical increase in progesterone during the luteal phase reduces LH pulse frequency, leading to diminished GnRH pulses, which are essential for regulating the menstrual cycle and sustaining pregnancy. A recent study challenges conventional beliefs, suggesting that higher progesterone levels before ovulation may trigger the LH surge, leading to follicle rupture and ovulation (140). Women with PCOS often face experience infrequent or absent ovulation due to low progesterone levels. Furthermore, hypothalamic feedback sensitivity to progesterone diminishes, impairing the suppression of LH (GnRH) pulses and resulting in ongoing gonadotropin secretion dysregulation (85). Therefore, progesterone emerges as a crucial component in the pathophysiology of PCOS.

### 8.1 The effect of natural compounds on progesterone in PCOS models

#### 8.1.1 Polyols

Research highlights the potential of polyols. Studies have shown that MYO supplementation can enhance luteal phase progesterone production in infertile women with PCOS, often restoring spontaneous fertility (141). Fedeli et al. (121) executed an animal study employing continuous light exposure on CD1 mice to establish a PCOS model. The results indicated that a 40:1 ratio of MYO to DCI could augment progesterone levels in PCOS-affected mice. This result was achieved by promoting the synthesis of CYP19A1 and FSHR, ultimately improving the fertility outcomes in these experimental models.

#### 8.1.2 Terpenoids

Research findings suggest that *β*-sitosterol may elevate progesterone levels in DHEA-induced PCOS mouse models by modulating gut microbiota, presenting a potential intervention for PCOS (86).

#### 8.1.3 Alkaloids

Evidence indicates that nicotinamide can boost progesterone levels in letrozole-induced PCOS rat models by activating AMPK expression, thus regulating the estrous cycle (126).

#### 8.1.4 Phenolics

Reddy et al. revealed that low and high doses of curcumin increased progesterone levels in letrozole-induced PCOS model rats, facilitating ovulation in a manner akin to CC. Subsequently, Shah et al. (96) noted that curcumin treatment might enhance progesterone concentrations through increased circulating adiponectin, thereby supporting ovulation and improving fertility. Kokabiyan et al. (99) reported that eugenol could elevate serum progesterone levels in EV-induced PCOS model rats by regulating the expression of the COX-2 and PPAR-α genes, thus enabling ovulation.

#### 8.1.5 Flavonoids

Khezri et al. (65) established a PCOS model in rats induced by EV, which led to reduced ovarian corpus luteum and progesterone levels. Treatment with genistein improved these changes, thus promoting follicular maturation. Similarly, Peng et al. (130) found that apigenin supplementation can enhance progesterone levels in DHEA-induced PCOS model rats, an effect potentially attributed to its ability to mitigate oxidative stress and suppress inflammatory cytokine expression, specifically TNF-α and IL-6. Mihanfar et al. (69) established a PCOS model using Wistar rats induced by letrozole administration. The study indicated that fisetin treatment could increase luteal progesterone concentrations. This effect was possibly due to enhanced antioxidant activity, as supported by elevated levels of CAT, SOD, and GPx, as well as increased SIRT1 and AMPK expression in the ovaries. Chahal et al. (142) recently published findings confirming fisetin’s role in treating PCOS by modulating AMPK/SIRT1 signaling pathways in rats.

#### 8.1.6 Organic acids

Chiang et al. (74) demonstrated that caffeic acid may enhance progesterone levels in rats by inhibiting the protein expression of 3β-HSD, leading to the restoration of ovarian morphology and estrous cycle normalization. Shalaby et al. (105) found that rosmarinic acid can increase progesterone levels in letrozole-induced PCOS rats, and this effect may be related to its inhibition of the gene expression of IL-1β, TNF-*α*, and VEGF in their ovarian tissue.

#### 8.1.7 Endogenous metabolites

Basheer et al. (136) established a PCOS model in Wistar rats using oral letrozole administration. Their findings indicated that melatonin treatment raises progesterone levels in rats by modulating steroidogenic enzyme activity (3β-HSD and 17β-HSD), thus restoring ovarian function in PCOS-affected rats.

#### 8.1.8 Vitamins

Research indicates that vitamin D supplementation can elevate progesterone levels in PCOS model rats affected by environmental factors, thereby facilitating ovarian development (78).

Overall, these compounds may increase progesterone production and restore ovulation by reducing oxidative stress or promoting the expression of related genes. Although natural compounds hold significant therapeutic potential for PCOS, research on their effects on progesterone levels is still limited, with most studies concentrating on the regulation of other hormones.

## 9 Estrogens in the pathogenesis of PCOS

Estrogens are primarily produced in the ovaries via two distinct pathways. These pathways involve converting androstenedione and testosterone into estrone and estradiol. These hormones are vital for female reproduction (143). Under normal physiological conditions, estradiol modulates gonadotropins via a dual feedback mechanism. During the late follicular phase, estradiol from the dominant follicle prompts a surge in LH, which is triggered by rapid GnRH pulses, ultimately inducing ovulation. Subsequently, estradiol provides negative feedback on FSH released by the pituitary, leading to the atresia of remaining follicles and ensuring the maturation of a single dominant follicle.

In PCOS, patients display impaired hypothalamic sensitivity to estradiol’s negative feedback. In addition, excess androgens can convert to estrogens in adipose tissue, increasing the-estrone-to-estradiol ratio (144). Long-term exposure of endometrial tissue to elevated estrogen levels may result in atypical hyperplasia and possibly endometrial carcinoma. In conclusion, estrogens play a critical role in the pathology of PCOS (145).

### 9.1 The effect of natural compounds on estrogens in PCOS models

#### 9.1.1 Polyols

A study by Fedeli et al. (121) found that a 40:1 combination of MYO and DCI can increase estradiol levels in a PCOS model maintained under continuous light exposure. This occurs through the regulation of androgen enzyme expression, along with increased synthesis of CYP19A1 and FSHR, thereby enhancing ovarian function.

#### 9.1.2 Terpenoids

Terpenoids exhibit considerable effects on PCOS models. Research indicates that crocetin enhances serum E2 levels in DHT-induced PCOS model mice by regulating the expression of AVPV-kisspeptin and ARC-kisspeptin, thereby restoring estrogen feedback mechanisms. Jin et al. (124) discovered that tanshinone IIA elevates serum estradiol levels in PCOS model mice induced by estradiol, likely through the modulation of FSHR and aromatase expression, thus improving ovarian function. All three of the following compounds can regulate estrogen homeostasis in DHEA-induced PCOS rodents. Bandariyan et al. (146) found that lutein, through its antioxidant properties, restored oocyte number and function in mice. Jujuboside A may promote CYP1A2 gene expression regulated by AhR (147). Finally, Ye et al. (92) demonstrated that ginsenoside compound K can stimulate CXCL14 gene expression, identifying it as a potential therapeutic target for PCOS.

#### 9.1.3 Polysaccharides

Polysaccharides and glycosides also play a role. The *Irpex lacteus* polysaccharide significantly raises estradiol levels in letrozole-induced PCOS rats, restoring ovarian histological morphology. However, the precise underlying molecular mechanisms remain unclear (54). Furthermore, trehalose feeding can reduce E2 levels in DHEA-induced PCOS model mice, possibly by regulating the ACE/AngII/AT1R pathway in the ovaries to alleviate oxidative stress and apoptosis in granulosa cells (55).

#### 9.1.4 Alkaloids

Yu et al. (58) conducted an *in vivo* investigation of letrozole-induced PCOS in rats, observing increased levels of E2. Their findings indicate that berberine treatment may normalize hormonal imbalances by modulating the PI3K/AKT signaling pathway, leading to ovarian structure restoration.

#### 9.1.5 Phenolics

Zhang et al. (148) found that resveratrol treatment elevated estradiol levels in letrozole-induced PCOS rats, which may be related to the regulation of adiponectin-1 protein levels and aromatase expression in ovarian tissue. Reddy et al. (128) found that high doses of curcumin in letrozole-induced PCOS model rats could restore the estradiol concentrations reduced by aromatase inhibitors, further validating the phytoestrogen properties of curcumin (149). Belani et al. (150) examined granulosa cells from patients with PCOS and revealed that swertiamarin could elevate estradiol levels in the conditioned medium of insulin-resistant PCOS individuals, offering potential benefits for this patient population. Shah et al. (60) further suggested that quercetin might reinstate aromatase activity in PCOS model mice by enhancing CYP19a1 and CYP11a1 expression, leading to increased estrogen levels in letrozole-induced PCOS model mice, thus presenting a viable treatment option.

#### 9.1.6 Flavonoids

Peng et al. (130) demonstrated that administering apigenin increases estradiol levels in DHEA-induced PCOS model rats, likely due to its antioxidant properties and the suppression of inflammatory cytokines such as TNF-*α* and IL-6. Huang and Zhang (68) established PCOS rat models using letrozole and a high-fat diet. After luteolin treatment, they observed an increase in serum E2 levels, possibly due to the activation of the Nrf2 pathway. Mihanfar et al. (69) found that fisetin increased E2 levels in letrozole-induced PCOS rats, likely due to elevated CAT, SOD, and GPx levels and reduced CYP17A1 expression. Wu et al. (70) found that naringenin caused the same changes in PCOS rats and proposed a positive correlation between estrogen and the gut microbiota. This points to the possibility that naringenin may improve PCOS-related endocrine disorders by modulating the gut microbiota.

#### 9.1.7 Organic acids

Shah et al. (71) identified that gallic acid may raise serum estrogen levels in letrozole-induced PCOS model mice by boosting the mRNA expression of CYP11a1 and CYP19a1. Research indicates that omega-3 polyunsaturated fatty acids can decrease E2 levels in DHEA-induced PCOS mouse models (103). In addition, chlorogenic acid has been shown to enhance estrogen levels in letrozole-induced PCOS Parkes mice, potentially linked to increased adiponectin levels (72). Chiang et al. (74) subcutaneously injected DHEA into SD rats to generate a PCOS model, resulting in excessive estrogen production in granulosa cells; their findings indicated that caffeic acid treatment can lower estrogen levels in rats, thus restoring normal estrous cycles. Pachymic acid can reduce E2 levels in PCOS model mice induced by DHEA, thereby improving the endocrine environment and oocyte quality of PCOS model mice (75). Research shows that rosmarinic acid increases E2 levels in letrozole-induced PCOS rats, mainly by reducing inflammation and angiogenesis (105).

#### 9.1.8 Endogenous metabolites

Basheer et al. (136) utilized letrozole to establish a PCOS model in Wistar rats. Their research demonstrated that melatonin treatment could enhance estradiol levels by modulating the activity of steroidogenic enzymes (3β-HSD and 17β-HSD), thereby fostering reproductive health in individuals affected by PCOS. Furthermore, Yu et al. (137) uncovered that melatonin upregulates CYP19A1 expression via the ERK pathway, resulting in increased estrogen levels in patients with PCOS, which promotes oocyte development.

#### 9.1.9 Vitamins

Studies have indicated that vitamin D supplementation can elevate estradiol concentrations in EV-induced PCOS model rats, thereby facilitating ovarian development (78).

As previously discussed, estrogen levels are typically elevated in PCOS patients, whereas they are often lower in PCOS animal models. This difference is influenced by the use of specific experimental inducers. For example, letrozole inhibits aromatase, thereby preventing estrogen synthesis and resulting in lower estrogen levels. This is a characteristic of the experimental model, not reflective of the natural state of PCOS. In animal models, researchers generally focus more on the mechanisms of androgen excess and ovulation disorders. Nevertheless, observing estrogen changes in animals treated with natural compounds remains valuable. Analyzing these changes helps us better understand the regulatory mechanisms, which can offer important insights for clinical treatment.

## 10 Prospects and conclusion

This article classifies natural compounds based on their chemical structures and biological sources, including polyols, terpenes, phenolics, flavonoids, polysaccharides, alkaloids, organic acids, endogenous metabolites, vitamins, and trace elements. It explores their effects on insulin, LH, FSH, androgens, estrogens, and progesterone, as well as their molecular and cellular mechanisms, which are crucial for understanding their therapeutic potential. Studies show that natural compounds regulate oxidative stress, apoptosis, signaling pathways, and protein gene expression through multiple targets, maintaining hormonal homeostasis and alleviating PCOS symptoms. Target molecules include CYP19A1, CYP11A1, CYP1A2, IRS-1, GLUT4, SIRT1, SIRT2, CXCL14, kisspeptin, CYP17A1, and Cox-2, while major signaling pathways include AMPK, PI3K/AKT, TGF-β1/Smad, JAK2/STAT3, HMGB1/TLR4/NF-κB, IRE1α-XBP1, and SIRT1/PGC-1α. In addition, natural compounds may regulate hormone levels in PCOS through mechanisms such as increasing adiponectin levels, reducing endoplasmic reticulum stress, and modulating the gut microbiota (Table 1).

**Table 1:** Effects and mechanisms of natural compounds on hormonal regulation in PCOS models.

Natural compounds	Structural classifications	Model	Related mechanisms	INS	LH	FSH	Androgen	Estrogen	P	References
Myo-inositol	Polyols	PCOS patients	None	✓	✓		✓		✓	(45–47, 141)
		Red blood cells in PCOS patients	Through phosphoinositide-related signal transduction and insulin-related metabolic response pathways							
D-chiro-inositol	Polyols	PCOS patients	None	✓			✓			(48)
Lupeol	Terpenoids	Mouse: DHEA-induced PCOS model	Regulating TLR-4 and TLR-2 gene expression, along with serum TNF-α levels; Through its antioxidant and anti-inflammatory effects	✓	✓		✓			(49, 90)
Astragaloside IV	Terpenoids	Rat: DHEA-induced PCOS model	By activating the PPARγ pathway and increasing autophagy levels	✓	✓		✓			(51)
Astaxanthin	Terpenoids	Infertile PCOS patients	None	✓						(52)
β-Sitosterol	Terpenoids	Mouse: DHEA-induced PCOS model	By modulating gut microbiota homeostasis		✓	✓			✓	(86)
Cryptotanshinone	Terpenoids	Rat: PCOS model induced by HCG and INS	Regulating the HMGB1/TLR4/NF-κB signaling pathway		✓		✓			(87)
Tanshinone IIA	Terpenoids	Mouse: E2-induced PCOS model	Regulating FSHR and aromatase expression				✓	✓		(124)
Thymoquinone	Terpenoids	Rat: letrozole-induced PCOS model	Increasing GPx1 gene transcription and reducing the expression of the Bax gene and the Bax/Bcl2 ratio		✓	✓	✓			(88)
Paeoniflorin	Terpenoids	Rat: DHEA-induced PCOS model	Modulating the TGF-β1/Smads signaling pathway		✓		✓			(89)
Crocetin	Terpenoids	Mouse: DHT-induced PCOS model	Regulating kisspeptin neurons		✓	✓	✓	✓		(91)
Ginsenoside compound K	Terpenoids	Rat: DHEA-induced PCOS model	Regulating CXCL14 expression in brown adipose tissue		✓		✓	✓		(92)
Nerolidol	Terpenoids	Rat: DHEA-induced PCOS model	Through its antioxidant effects		✓	✓	✓			(93)
Artemisinins	Terpenoids	Rat: DHEA-induced PCOS model	Directly targeting LONP1 inhibits CYP11A1 levels				✓			(122)
Mogroside V	Terpenoids	Rat: letrozole- and High-fat diet-induced PCOS model	Increasing the expression of LDHA, HK2, and PKM2				✓			(123)
Lutein	Terpenoids	Mouse: DHEA-induced PCOS model	Through its antioxidant activity					✓		(146)
Jujuboside A	Terpenoids	Mouse: DHEA-induced PCOS model	Activation of AhR regulates CYP1A2 expression					✓		(147)
Coenzyme Q10	Terpenoids	PCOS patients	None	✓						(50)
Dendrobium nobile polysaccharide	Polysaccharides	Rat: letrozole- and high-fat diet-induced PCOS model	Activating SIRT2	✓						(53)
Irpex lacteus polysaccharide	Polysaccharides	Rat: letrozole-induced PCOS model	Enhancing antioxidant enzyme expression and inhibiting the TGF-β1/Smad pathway	✓	✓		✓	✓		(54)
Trehalose	Polysaccharides	Mouse: DHEA- and High-fat diet-induced PCOS model	Improving oxidative stress and apoptosis in ovarian granulosa cells by downregulating ACE/AngII/AT1R	✓			✓	✓		(55)
Berberine	Alkaloids	Rat: DHEA-induced PCOS model	Inhibiting apoptosis and regulating key signaling molecules (TLR4, LYN, PI3K, AKT, NF-kB, TNF-α, IL-1, IL-6, caspase-3)	✓	✓		✓	✓		(56–58, 125)
		Rat: letrozole-induced PCOS model	Mediating via PI3K/AKT pathway							
Nicotinamide	Alkaloids	Rat: letrozole-induced PCOS model	Downregulating CYP17A1 gene expression and activating AMPK expression				✓		✓	(126)
Resveratrol	Phenolics	Rat: letrozole- and High-fat diet-induced PCOS model	Regulating SIRT2	✓	✓	✓	✓	✓		(59, 94, 111, 148)
		Rat: PCOS model based On TBT exposure	Facilitating calcium ion transport and activating CaMKII β to repair projections							
		Rat: letrozole-induced PCOS model	Regulating nesfatin-1 protein levels and aromatase expression in ovarian tissue							
		PCOS patients	Influencing gene expression of VEGF and HIF1 in particle cells							
Quercetin	Phenolics	Mouse: letrozole-induced PCOS model	Reducing plasma vascular endothelial growth factor levels and increasing the expression of CYP19A1 and CYP11A1	✓	✓	✓	✓	✓		(60, 61, 95)
		PCOS patients	Increasing adiponectin levels							
		Rat: testosterone-induced PCOS model	Inhibiting PI3K to suppress CYP17A1 gene expression							
Trans-Anethole	Phenolics	Rat: testosterone-induced PCOS model	Related to its antioxidant capacity and protective effect on liver and kidney tissues	✓			✓			(62, 129)
Curcumin	Phenolics	Rat: DHEA-induced PCOS model	Inhibiting the IRE1 α-XBP1 pathway and activating the PI3K/AKT signaling pathway		✓	✓	✓	✓	✓	(96, 97, 127, 128)
		Mouse: letrozole-induced PCOS model	Regulating the balance of circulating Testosterone and adiponectin							
		Rat: letrozole-induced PCOS model	Regulating the PTEN and IRS1/PI3K/GLUT4 pathways							
		PCOS patients	None							
Rhamnocitrin	Phenolics	Rat: letrozole-induced PCOS model	Through its antioxidant activity; increasing PPAR - γ activity in the ovaries and inhibited the TGF-β 1/Smad pathway		✓	✓	✓			(98)
Eugenol	Phenolics	Rat: EV-induced PCOS model	Regulating the expression of Cox-2 and PPAR-α genes		✓	✓	✓		✓	(99)
Swertiamarin	Phenolics	Granulocytes in PCOS patients	None					✓		(150)
Total flavonoids	Flavonoids	Rat: DHEA-induced PCOS model	Regulating the IL-6 mediated JAK2/STAT3 signaling pathway	✓	✓	✓	✓			(64)
Genistein	Flavonoids	Rat: EV-induced PCOS model	None	✓	✓	✓			✓	(65–67)
Luteolin	Flavonoids	Rat: letrozole- and high-fat diet-induced PCOS model	Regulating the PI3K/AKT/Nrf2 signaling pathway	✓	✓	✓	✓	✓		(68)
Fisetin	Flavonoids	Rat: letrozole-induced PCOS model	Increase expression of SIRT1 and AMPK in ovaries; Reducing CYP17A1 expression; through its antioxidant activity	✓			✓	✓	✓	(69, 142)
Naringenin	Flavonoids	Rat: letrozole-induced PCOS model	Modulating gut microbiota and the SIRT1/PGC-1ɑ signaling pathway	✓	✓	✓	✓	✓		(70)
Silibinin	Flavonoids	Rat: letrozole-induced PCOS model	Through its antioxidant and anti-inflammatory properties		✓		✓			(100)
Baicalin	Flavonoids	Rat: DHEA-induced PCOS model	Regulating the expression of miR-874-3p/FOXO3 and miR-144/FOXO1 genes		✓		✓			(101)
Myricetin	Flavonoids	Mouse: DHEA-induced PCOS model	Activating brown adipose tissue		✓					(102)
Apigenin	Flavonoids	Rat: DHEA-induced PCOS model	Exerting antioxidant activity and suppressing the expression of inflammatory cytokines (TNF-α and IL-6)				✓	✓	✓	(130)
Puerarin	Flavonoids	PCOS patients	Increasing levels of SHBG and SOD in the blood				✓			(131)
Rutin	Flavonoids	Rat: letrozole-induced PCOS model	Through its antioxidant activity				✓			(132)
Gallic acid	Organic acids	Mouse: letrozole-induced PCOS model	Regulating Adipo R1 and adiponectin expression; Enhancing CYP11a1 and CYP19a1 mRNA levels; Boosting ovarian antioxidant capacity	✓		✓	✓	✓		(71)
Chlorogenic acid	Organic acids	Mouse: letrozole-induced PCOS model	Regulating adiponectin receptors and their antioxidant capacity	✓	✓	✓	✓	✓		(72)
Omega-3 fatty acids	Organic acids	PCOS patients Mouse: DHEA-induced PCOS model	Increasing adiponectin levels suppressing ovarian inflammation	✓	✓		✓	✓		(73, 103)
Caffeic acid	Organic acids	Rat: DHEA-induced	Alleviating endoplasmic reticulum stress	✓			✓	✓	✓	(74)
		PCOS model	Oxidative stress, and inhibiting the expression of 3β-HSD protein							
Pachymic acid	Organic acids	Mouse: DHEA-induced PCOS model	Regulating CYP-17, IRS-1, and GLUT4 expression and their anti-inflammatory effects	✓			✓	✓		(75)
Sinapic acid	Organic acids	Rat: letrozole-induced PCOS model	Through its antioxidant activity		✓	✓	✓			(104)
Rosmarinic acid	Organic acids	Rat: letrozole-induced PCOS model	Through its anti-inflammatory and anti-angiogenic effects		✓		✓	✓	✓	(105)
γ-Linolenic acid	Organic acids	Rat: DHEA-induced PCOS model	Through its anti-inflammatory effects				✓			(133)
Melatonin	Endogenous metabolites	PCOS patients	Through the ERK pathway	✓			✓	✓	✓	(76, 135–137)
		Rat: letrozole-induced PCOS model	Through the modulation of steroidogenic enzyme activity							
Acetyl-L-carnitine	Endogenous metabolites	PCOS patients	None	✓	✓					(77)
Vitamin D	Vitamins	Rat: EV-induced PCOS model	None	✓	✓	✓	✓	✓	✓	(78, 112)
Vitamin E	Vitamins	PCOS patients	None				✓			(134)

LH, luteinizing hormone; FSH, follicle-stimulating hormone; P, progesterone; FSHR, follicle-stimulating hormone receptor.

The analysis shows that different types of natural compounds selectively affect specific hormones. For example, terpenes are more effective in regulating androgens, while flavonoids are particularly effective in modulating insulin and LH. These properties highlight the targeted therapeutic potential of natural compounds. Future research could explore these compounds further to develop more effective treatments for PCOS.

Natural compounds are an important source for clinical drug research, but their application in PCOS still faces several challenges. Despite positive results in animal studies, translating these findings into effective clinical treatments remains a challenge, especially in determining standardized dosages and treatment durations, where individual differences must be considered. In addition, the safety and long-term effects of some natural compounds have yet to be fully verified and require further clinical data. Future research should capitalize on the multi-target advantages of natural compounds, explore their mechanisms of action in greater depth, and investigate their potential for combination with conventional drugs. Mechanisms such as oxidative stress, inflammation, and IR play a central role in both PCOS and many other metabolic and inflammatory diseases. Therefore, interdisciplinary research across these diseases could integrate existing therapeutic strategies and provide new insights. Moreover, the regulatory effects of natural compounds on the gut microbiota and other PCOS-related hormones, such as anti-Klebsiella toxin, adiponectin, and inhibin, should be further explored. Through continued research, we aim to develop treatments with fewer side effects and greater efficacy, offering new therapeutic options for PCOS patients.
